# Nationwide Estimates of Viral Load Suppression and Acquired HIV Drug Resistance in Cameroon

**DOI:** 10.1016/j.eclinm.2018.06.005

**Published:** 2018-07-04

**Authors:** Gaëlle F. Tchouwa, Sabrina Eymard-Duvernay, Amandine Cournil, Nadine Lamare, Laetitia Serrano, Christelle Butel, Silvia Bertagnolio, Eitel Mpoudi-Ngole, Elliot Raizes, Avelin F. Aghokeng, EHRICA Study Group

**Affiliations:** a Centre de Recherche sur les Maladies Emergentes et Reemergentes (CREMER), Virology laboratory IMPM-IRD, IMPM, Yaoundé, Cameroon; b Institut de Recherche pour le Développement (IRD) UMI 233, INSERM U1175, Université de Montpellier, Unité TransVIHMI, Montpellier, France; c HIV Department, World Health Organization, Geneva, Switzerland; d Centers for Disease Control and Prevention, Atlanta, GA, USA

**Keywords:** HIV, Viral load suppression, Drug resistance, Cameroon

## Abstract

**Background:**

Population-based studies to estimate viral load (VL) suppression and rate of acquired HIV drug resistance (ADR) are essential in sub-Saharan Africa. We conducted the first nationally representative study estimating VL suppression and ADR in Cameroon.

**Methods:**

Eligible participants were patients on antiretroviral therapy (ART) for 12 to 24 months (ART 12–24) or 48 to 60 months (ART 48–60). ART 12–24 participants were recruited from 24 randomly selected clinics in both urban and rural regions. ART 48–60 participants were recruited from 7 urban clinics. Recruitment occurred from February to August 2015. Dried blood spots (DBSs) and plasma specimens were collected and tested for HIV-1 RNA level and presence of drug resistance mutations (DRM) when VL ≥ 1000 copies/ml.

**Results:**

Overall, 1064 ART 12–24 and 388 ART 48–60 participants were recruited. Viral suppression in the ART 12–24 group was 72.1% (95% CI: 66.3–77.2) overall, 75.0% (65.2–82.7) in urban sites, and 67.7% (58.3–75.8) in rural sites. In the ART 48–60 group, viral suppression was 67.7% (55.8–77.7). Overall, HIV drug resistance (HIVDR) was 17.7% (15.1–20.6) and 28.3% (17.4–42.5) in the ART 12–24 and ART 48–60 groups, respectively. However, among patients with VL ≥ 1000 copies/ml, HIVDR was identified in 63.3% (52.0–73.3) of ART 12–24 patients, and in 87.7% (67.4–96.1) of ART 48–60 patients.

**Conclusions:**

Results of this first nationwide study indicate alarming levels of virological failure and ADR in Cameroon. Better ART management is urgently needed and should focus on improving ART adherence, availability of VL monitoring, and more timely switches to second-line ART.

**Research in context**

**Evidence before this study**

Many studies have looked at viral load suppression and HIV drug resistance amongst individuals on ART in Cameroon and they have generally described populations from urban regions, generally from the two major cities, Yaoundé and Douala. These studies generally reported 10% to 20% virological failure in populations on ART for 12 to 24 months. We searched PubMed using the following terms, “hiv*” AND “antiretroviral” AND “drug resistance”, restricting to English language with no publication date restriction, on July 4, 2017. No studies that we could identify have assessed virological outcome and HIV drug resistance at the national level in Cameroon or in other low- and middle-income countries. A few studies have estimated the long-term virological outcome and outcome amongst population from the rural regions.

**Added value of this study**

We show that current national levels of early and late virological failure amongst individuals on first-line ART in Cameroon are substantially high. Populations form rural regions are also at higher risk of failure with subsequent development of drug resistance.

**Implication of all the available evidence**

Strategies to improve ART management are urgently needed and should include a better availability of viral load monitoring, and timely switches to second-line ART. Also, more robust first-line drugs should be considered in the developing countries to prevent the rapid emergence of drug resistant HIV.

Alt-text: Unlabelled Box

## 1 Introduction

Thirty years in the global fight against the acquired immune deficiency syndrome (AIDS) caused by the human immunodeficiency virus (HIV) have provided critical lessons and important insights concerning the origin, epidemiology and evolution of this infection. While the fight is still far from ended, currently available tools and strategies to combat and/or control the infection have been significantly improved over the past thirty years. The Joint United Nations Programme on HIV/AIDS (UNAIDS) has established the goal of ending the AIDS epidemic by 2030 [1]. This UNAIDS strategy includes several important sub-objectives, one of which is to reach “90% of virological suppression in all people receiving antiretroviral therapy” by 2020. Indeed, access to antiretroviral therapy (ART) is the key element of this strategy, not only to treat and control the infection in infected persons, but also as a tool to prevent new infections [2]. While historically the focus has been on rapid scale-up of ART, and approximately 18 million individuals are now receiving ART globally, is now increasingly important to assess whether those receiving ART achieve viral load (VL) suppression and the emergence of HIV drug resistance (HIVDR) among individuals failing ART.

Acquired HIV drug resistance (ADR) emerges under drug pressure and can significantly impair the efficacy of antiretrovirals (ARV), and thus represents a major risk for the efficacy of national ART program. Moreover, an increased prevalence of drug-resistant HIV could precipitate a future HIV epidemic driven by drug resistant strains. To prevent ADR, it is critical to rapidly achieve sustained viral suppression after ART initiation, and thus avoid premature failures and switches [3]. ADR is of particular concern in the context of sub-Saharan Africa. In this region, drugs with low genetic barriers to resistance, mostly nucleoside reverse transcriptase inhibitors (NRTI) and non-nucleoside reverse transcriptase inhibitors (NNRTI), are widely used and drug resistance mutations (DRM) to these drug classes (such as M184V, K103N, and Thymidine Analogue Mutations (TAMs)) are reported at high frequency, both in children and in adults [4], [5]. Owing to the limited availability of VL monitoring and subsequent delayed switching to second-line ART, accumulation of DRMs is frequently reported and represents a major challenge in the region [6], [7], [8].

Health related outcomes on ART among people living with HIV in low- and middle-income settings, such as in sub-Saharan Africa, have raised a number of significant concerns and challenges because of the high number of people needing ART and operational difficulties. Studies conducted in different sub-Saharan African countries report variable virological outcomes in patients on ART, with low to very high rates of virologic failure (VF) reported [9], [10], [11], [12], [13], [14]. Variation in virological outcomes is also observed within countries, mostly because of different study designs, different population characteristics and limited generalizability of data. To inform national and international stakeholders, and provide reliable data to guide decision-making, it is critical to assess, in a standardized and nationally representative manner, VL suppression and ADR. The following study is the first national evaluation of VL suppression and HIVDR in Cameroon.

## 2 Material and Methods

### 2.1 Study Sites and Participants

The study was a cross sectional investigation, implemented in Cameroon in February 2015. The methodology is based on the WHO-recommended two-stage cluster design using probability proportional to proxy size (PPPS) sampling [15]. In this approach, two treatment time points are investigated and include populations on ART for 12 to 24 months and those on ART for 48 to 60 months. The 12–24 months assessment aims at evaluating virological failure during the first years of ART initiation, and the 48–60 months assessment evaluates the outcome later after ART initiation.

#### 2.1.1 ADR Survey Among Individuals on ART for 12 to 24 Months (ART 12–24)

A total of 29 ART clinics (19 urban clinics with one clinic sampled two times and 10 rural clinics) were sampled with urban/rural stratification, from a country-wide listing of all 154 clinics providing ART. The respective number of patients on ART at the end of 2014 in each clinic was provided to estimate clinic size. In PPPS sampling, clinics are sampled proportionally to the total number of patients on ART in each clinic. Thus, clinics with a larger number of patients on ART are more likely to be sampled than smaller clinics [15]. The required sample size (N = 960, 32 participants per clinic) was calculated to obtain an estimate of drug resistance with a confidence interval (CI) of ± 5%, assuming a drug resistance prevalence of 11%, genotyping failure rate of 15%, an intra-class correlation set to 0.1 and a design effect due to imperfect weighting information set to 1.5.

#### 2.1.2 ADR Survey Among Individuals on ART for 48 to 60 Months (ART 48–60)

For this survey, we selected ART clinics with more than 48 months activity to maximize the probability of obtaining the required sample size per clinic. A total of 10 clinics were eligible, all in urban regions. The required sample size (N = 400 participants, 40 per clinic) was calculated to obtain an estimate of drug resistance with a CI of ± 5%, assuming a drug resistance prevalence of 18%, genotyping failure rate of 15%.

ART 12–24 and ART 48–60 participants were consecutively recruited in selected study clinics if they were HIV-1 positive, aged ≥ 18 years, were still on ART, provided written informed consent and had been on ART for 12 to 24 months (ART 12–24 population) or for 48–60 months (ART 48–60 population). Socio-demographic and clinical data were collected using a questionnaire, which included participant age, gender, date of ART start, and ongoing ART regimen.

The study protocol was approved by the Associate Director for Science in the Center for Global Health at the Centers for Disease Control and Prevention for non-research determination (Atlanta, USA) and the Cameroon National Ethics Committee for Health Research (Yaoundé, Cameroon).

### 2.2 Laboratory Methods

Freshly collected whole blood was used to prepare dried blood spots (DBSs) according to previously published standards [16]. Three DBS cards were prepared for each participant and sent to the reference national laboratory for HIVDR genotyping. One DBS card was used for initial laboratory analyses (viral load and HIVDR testing), a second DBS card was used as a back-up for re-testing was necessary, and the last card was sent to an external reference laboratory for quality control. HIV-1 RNA quantification was performed on DBS using the Abbott *m*2000rt RealTi*me* HIV-1 kit according to manufacturer recommendations (Abbott Pack, IL, USA). All DBS samples with VL ≥ 1000 copies/ml were considered for HIV-1 drug resistance genotyping. Nucleic acids were extracted using the *m*2000rt method. The viral protease (PR) and reverse transcriptase (RT) regions were separately amplified to optimize the polymerase chain reaction (PCR) outcome as previously published [16]. A one-step RT-PCR was performed with primers PR2 (5′-CCTAGRAAAARGGGCTGTTGGAAATGT-3′, forward) and TR2as (5′-AATYTGACTTGCCCARTTTARTTTTCC-3′, reverse). Separated nested-PCRs were performed in the PR region (amino acids 1–99) using PR3 (5′-GARGGACAYCAAATGAAAGAYTGYAC-3′) and PR3as (5′-GCCATTGTTTAACYTTTGGDCCATCCATT-3′), and in the RT region (amino acids 1–260) with TR3 (5′-TGATAGGRGGAATTGGAGGTTTTATCAA-3′) and TR3as (5′-CTAAYTTYTGTATRTCATTGACAGTCCA-3′). RT-PCRs were carried out with 10 to 15 μl of the RNA extracts using the Qiagen one-step RT-PCR kit (Qiagen, Courtaboeuf, France). Five microliters of the RT-PCR product were used for nested PCR using the HotStartTaq master mix kit (Qiagen, Courtaboeuf, France). PCR products were purified and directly sequenced using the BigDye Terminator v3.1 Cycle Sequencing kit (Applied Biosystems, Carlsbad, CA). DRM in PR and RT were identified using the Stanford interpretation algorithms, version 8.3 (https://hivdb.stanford.edu/).

### 2.3 Statistical Analysis

The expected outcome of the study was to generate population-level prevalence for VL suppression and HIVDR for each of the study time points. VL suppression was defined as a classified VL < 1000 copies/ml. Prevalence of each outcome was calculated as a ratio, where the denominator is an estimate of the number of eligible individuals in the country during the survey period and the numerator is an estimate of such individuals with the outcome of interest. Prevalence of VL suppression was estimated among the total eligible population as well as for the sub-populations of individuals on first-line ART. For drug resistance outcome, overall prevalence of drug resistance was estimated as the proportion of individuals with any drug resistance among the total eligible population including individuals with both VL < and ≥ 1000 copies/ml, but excluding individuals with unsuccessful genotypes. Prevalence of any, protease inhibitor (PI), NRTI and NNRTI drug resistance were also estimated among individuals with VL ≥ 1000 copies/ml with successful genotyping. Estimates were given for the total population in both surveys and according to urban and rural stratum for the ART 12–24 survey. Proportions and means were estimated using “*svy*” Stata commands (Stata 14, StataCorp, College Station, TX, USA) to take into account the stratified two-stage cluster design of the ART 12–24 samples and ART 48–60 samples, respectively. Sampling weights accounting for probability of selection at each stage (clinic, patient) and non-response were defined for all outcomes. Taylor linearization method was used to estimate standard errors. A finite population correction was applied. Ninety-five percent CIs were calculated using a logit transformation. Intra-class correlation, i.e. proportion of outcome's total variance that is shared within clinics, was estimated for ADR prevalence outcome using analysis of variance.

### 2.4 Sequence Accession Number

The newly reported protease and reverse transcriptase sequences are available in GenBank under the following accession numbers: MF797024–MF797285.

### 2.5 Role of the Funding Source

The funder of the study had no role in study design, data collection, data analysis, data interpretation, or writing of the report. The corresponding author had full access to all the data in the study and had final responsibility for the decision to submit for publication.

## 3 Results

### 3.1 Participants' Characteristics

**Fig. 1:**
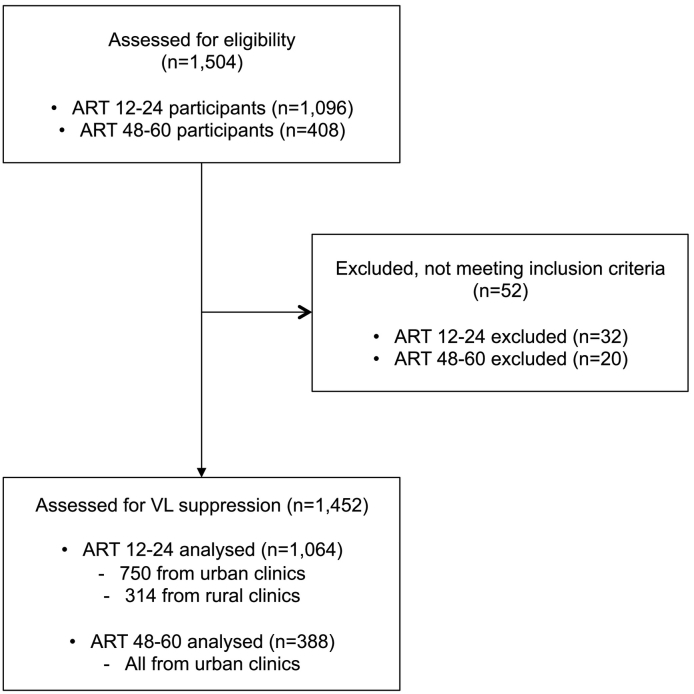
Flow chart of participants in the study. ART 12–24 represents participants on ART for 12 to 24 months. ART 48–60 represents participants on ART for 48 to 60 months.

For participants on ART for 12 to 24 months (ART 12–24 population), 1096 individuals were recruited in 24 clinics out of 29. Five clinics, located in the northern region were excluded due to Boko Haram attacks and the related security issues. Among these 5 clinics, 3 were ART 48–60 recruitment clinics. Additional patients were recruited in the remaining 24 clinics to achieve the required number of participants as recommended [15]. One thousand and sixty-four eligible participants ultimately considered for the assessment after 32 were excluded because they did not meet the eligibility criteria (Fig. 1). Among these participants, 750 and 314 were recruited in urban and rural clinics, respectively. For participants who had been on ART for 48 to 60 months (ART 48–60 population), after exclusion of 20 ineligible participants, 388 individuals, all from urban clinics, were included in the analysis. In both study populations, women predominated (Fig. 1). The median time on ART was 17 months for ART 12–24 participants, similar in both rural and urban clinics, and 53 months for ART 48–60 participants. Predominant ARV regimens at inclusion in the study were tenofovir (TDF) + lamivudine (3TC)/emtricitabine (FTC) + efavirenz (EFV)/nevirapine (NVP) and zidovudine (AZT) + 3TC + EFV/NVP (Table 1). Lopinavir (LPV)-based regimens, essentially 2NRTIs + LPV/r, represented 1.1% and 5.4% of ARV regimens in the ART 12–24 and ART 48–60 groups respectively. The median time delays between DBS collection in the clinics and their shipment and storage at − 20 °C to − 30 °C in the central laboratory ranged from 9 to 13 days (Table 1).

**Table 1:** Study participants and characteristics.

Study participants	ART 12–24						ART 48–60	
	Urban clinics		Rural clinics		Total		Urban clinics	
	n	Estimates^a^	n	Estimates^a^	n	Estimates^a^	n	Estimates^a^
Total participants recruited	776		320		1096		408	
Not eligible (excluded)	26		6		32		20	
Total eligible for the study	750		314		1064		388	
Gender ratio, female	563	77.7% (73.8–81.3)	245	78.1% (75.2–80.8)	808	77.9% (75.4–80.2)	287	75.3% (66.6–82.3)
Age, years		39.5 (38.8–40.3)		40.8 (39.3–42.3)		40.0 (39.4–40.7)		43.1 (42.0–44.3)
ARV regimens								
TDF + 3TC + EFV/NVP	642	84.5% (78.7–89.0)	225	74.3% (56.0–86.8)	867	80.5% (74.3–85.4)	287	75.0% (60.4–85.5)
AZT + 3TC + EFV/NVP	92	13.9% (9.6–19.8)	88	25.2% (13.0–43.2)	180	18.4% (13.7–24.3)	77	19.6% (10.2–34.3)
2NRTIs + LPV/r	15	1.6% (0.7–3.3)	1	0.4% (0.0–4.4)	16	1.1% (0.6–2.2)	23	5.4% (1.8–15.5)
Time DBS spent on site, days		10.8 (6.4–15.1)		13.0 (11.7–14.3)		11.7 (9.1–14.2)		8.9 (5.8–11.9)

ARV: antiretroviral drug; TDF: tenofovir; 3TC: lamivudine; EFV: efavirenz; NVP: nevirapine; AZT: zidovudine; FTC: emtricitabine; LP/r: boosted Lopinavir; NRTI: nucleoside reverse transcriptase inhibitor.

a Study-design weighted proportion (95% CI) or study-design weighted mean (95% CI).

### 3.2 Prevalence of VL Suppression

All samples from eligible participants were tested in both ART 12–24 (n = 1064) and ART 48–60 (n = 388) groups. In the ART 12–24 group, VL suppression prevalence was 72.1% (95% CI: 66.3–77.2) overall; 75.0% (65.2–82.7) and 67.7% (58.3–75.8) in urban and rural clinics respectively. A sub-analysis including only ART 12–24 participants on first-line NNRTI-based ART showed similar results: VL suppression was 72.1% (66.5–77.2) overall; 75.2% (65.7–82.7) and 67.5% (58.0–75.8) in urban and rural clinics respectively (Table 2). In ART 48–60 population, VL suppression was 67.7% (55.8–77.7) overall, and 68.7% (56.0–79.1) in participants on first-line NNRTI-based regimen (Table 2). The estimated intra-class correlation, providing an indication of similarity of participants within the sites for the VL suppression outcome, was 0.07 (95% CI: 0.02–0.13) and 0.08 (95% CI: 0.00–0.19) for ART 12–24 and ART 48–60 surveys, respectively.

**Table 2:** VF and drug resistance rates after 12–24 months and 48–60 months on ART.

*Virological outcome (VL copies/ml)*	ART 12–24						ART 48–60	
	Overall		Urban sites		Rural sites		Urban sites	
	n/N	Estimates^a^	n/N	Estimates^a^	n/N	Estimates^a^	n/N	Estimates^a^
*Total VL performed*	*1065*		*750*		*315*		*391*	
*Total valid VL results*	*1064*		*750*		*314*		*388*	
VL < 1000, (95% CI)	796/1064	72.1% (66.3–77.2)	590/750	75.0% (65.2–82.7)	206/314	67.7% (58.3–75.8)	267/388	67.7% (55.8–77.7)
VL < 1000 in on first-line ART, (95% CI)	786/1050	72.1% (66.5–77.2)	581/737	75.2% (65.7–82.7)	205/313	67.5% (58.0–75.8)	255/364	68.7% (56.0–79.1)
Mean VL (Log10) for VL > 1000 copies/ml		4.37 (4.18–4.56)		4.39 (4.14–4.64)		4.35 (3.99–4.71)		4.35 (4.14–4.57)
*Total genotypes performed*	*268*		*160*		*108*		*121*	
*Total successfully genotyped*	*164*		*105*		*59*		*67*	
Overall HIVDR %, (95% CI)	113/960	17.7% (15.1–20.6)	70/695	14.0% (11.3–17.3)	43/265	23.2% (16.6–31.5)	59/334	28.3% (17.4–42.5)
HIVDR % in VL ≥ 1000								
Any DRM, (95% CI)	113/164	63.3% (52.0–73.3)	70/105	56.1% (42.7–68.6)	43/59	71.7% (54.5–84.3)	59/67	87.7% (67.4–96.1)
PI DRM, (95% CI)	2/164	2.3% (0.7–7.2)	1/105	2.3% (0.6–9.3)	1/59	2.3% (0.2–19.0)	1/67	0.8% (0.1–9.4)
NRTI DRM, (95% CI)	97/164	52.6% (41.2–63.7)	59/105	49.1% (37.0–61.4)	38/59	56.6% (34.3–76.5)	53/67	77.9% (50.2–92.5)
NNRTI DRM, (95% CI)	111/164	61.9% (50.8–71.9)	69/105	55.5% (42.7–67.6)	42/59	69.4% (50.6–83.4)	59/67	87.7% (67.4–96.1)

VL: viral load; CI: confidence interval; HIVDR: HIV drug resistance; DRM: drug resistance mutation; PI: protease inhibitor; NRTI: nucleoside reverse transcriptase inhibitor; NNRTI: non-nucleoside reverse transcriptase inhibitor.

a Study-design weighted proportion (95% CI) or study-design weighted mean (95% CI).

### 3.3 Prevalence of HIV-1 Drug Resistance

All samples with VL ≥ 1000 copies/ml underwent further drug resistance genotyping. This included 268 samples for the ART 12–24 group, 160 and 108 from urban and rural sites respectively, and 121 samples for the ART 48–60 group. Genotyping success rate increased in DBS with high VL and we observed more genotyping failures in samples with low VL. Correlation analyses showed that mean VL was 3.6 Log10 in DBS that failed genotyping for the two study groups. For DBS successfully genotyped, mean VL were 4.8 Log10 and 4.9 Log10 for ART 12–24 and ART 48–60, respectively. Overall, genotyping failure rate was higher that 15%. HIV-1 subtypes included CRF02-AG (155/231), A1 (22), G (17), F2 (10), CRF11_cpx (7), D (5), CRF01_AE (2), CRF13_cpx (2), CRF18_cpx (2), B (1), CRF06_cpx (1), CRF09_cpx (1), other recombinant forms (6). Crude proportions of VL suppression and HIVDR in each site are showed in Supplementary Table 1. An overall prevalence of drug resistance was estimated as the proportion of individuals with any DR among the total eligible population including individuals with both VL < and ≥ 1000 copies/ml, but excluding individuals with unsuccessful genotypes. The overall HIVDR rate was 17.7% (15.1–20.6) in the ART 12–24 group. In urban clinics, the overall HIVDR rate was 14.0% (11.3–17.3) and that rate was significantly higher in rural clinics, 23.2% (16.6–31.5). In the ART 48–60 group, HIVDR rate was 28.3% (17.4–42.5) overall (Table 2).

In the study participants experiencing VF, i.e. VL ≥ 1000 copies/ml, HIVDR rates were 63.3% (52.0–73.3) and 87.7% (67.4–96.1) in ART 12–24 and ART 48–60 populations respectively. The estimated intra-class correlation for the HIVDR outcome, was 0.08 (95% CI: 0.00–0.20) and 0.06 (95% CI: 0.00–0.25) for ART 12–24 and ART 48–60 surveys, respectively. In urban and rural clinics from the ART 12–24 group, HIVDR was detected in 56.1% (42.7–68.6) and in 71.7% (54.5–84.3) of the failures, respectively. Resistance to PI drugs represented 2.3% (0.7–7.2) and 0.8% (0.1–9.4) in the ART 12–24 and ART 48–60 groups respectively. Resistance to NRTI drugs represented 52.6% (41.2–63.7) and 77.9% (50.2–92.5) in the ART 12–24 and ART 48–60 groups respectively. For NNRTI drug resistance, 61.9% (50.8–71.9) and 87.7% (67.4–96.1) rates were observed in the ART 12–24 and ART 48–60 groups respectively (Table 2). In the ART 12–24 urban sites, resistance to drug classes were 2.3% (0.6–9.3), 49.1% (37.0–61.4), and 55.5% (42.7–67.6) for PIs, NRTIs and NNRTIs respectively. In the ART 12–24 rural sites, resistance to drug classes were 2.3% (0.2–19.0), 56.6% (34.3–76.5), and 69.4% (50.6–83.4) for PIs, NRTIs and NNRTIs respectively (Table 2).

### 3.4 DRMs and Mutation Profiles

In the ART 12–24 group, no major PI DRM was found. In this group, predominant NRTI DRMs included M184VI, 50.2% (40.0–60.3); K65R, 16.4% (10.1–25.5); T215FY, 10.2% (5.9–17.1); and K219QE, 8.8% (5.7–13.3) (Table 3). Predominant NNRTI DRMs were K103N, 32.0% (24.8–40.1); Y181CIV, 18.7% (12.6–26.9); G190ASEQ, 15.9% (11.0–22.3); and Y188LCH, 5.4% (1.9–14.3). In the ART 48–60 group, one participant carried a virus with a major PI DRM, N88S. Predominant NRTI DRMs included M184VI, 73.1% (55.1–85.7); T215FY, 25.0% (12.3–44.2); K65R, 20.4% (8.7–40.7), and K219QE, 16.7% (7.3–33.7). Predominant NNRTI DRMs included K103N, 50.1% (38.6–61.5); Y181CIV 25.0% (18.7–32.7); G190ASEQ, 16.7% (9.1–28.6); and Y188LCH, 16.1% (8.7–27.9).

**Table 3:** Predominant HIV-1 drug resistance mutations.

	ART 12–24 (n = *164*)		ART 48–60 (n = *67*)	
	n	Estimates (95% CI)^a^	n	Estimates (95% CI)^a^
PI DRMs				
N88S	0	–	1	0.8% (0.1–9.4)
NRTI DRMs				
M184VI	91	50.2% (40.0–60.3)	50	73.1% (55.1–85.7)
K65R	32	16.4% (10.1–25.5)	13	20.4% (8.7–40.7)
T215FY	18	10.2% (5.9–17.1)	16	25.0% (12.3–44.2)
K219QE	19	8.8% (5.7–13.3)	9	16.7% (7.3–33.7)
NNRTI DRMs				
K103N	60	32.0% (24.8–40.1)	37	50.1% (38.6–61.5)
Y181CIV	34	18.7% (12.6–26.9)	17	25.0% (18.7–32.7)
G190ASEQ	30	15.9% (11.0–22.3)	12	16.7% (9.1–28.6)
Y188LCH	11	5.4% (1.9–14.3)	8	16.1% (8.7–27.9)

DRM: drug resistance mutation; PI: protease inhibitor; NRTI: nucleoside reverse transcriptase inhibitor; NNRTI: non-nucleoside reverse transcriptase inhibitor.

a Study-design weighted proportion (95% CI) or study-design weighted mean (95% CI).

Several mutation profiles were identified, ranging from a single mutation to complex profiles with up to nine major DRMs (Supplementary Table 2). K103N and K103N + M184V profiles predominated in both ART 12–24 and ART 48–60 groups. Except for K103N, all single mutations were found only in the ART 12–24 group and included V106A, G190A, and T215I. The other DRM profiles included up to 9 mutations and were found in both ART 12–24 and ART 48–60 groups (Supplementary Table 2).

## 4 Discussion

In their fight against HIV/AIDS, Cameroon, like many other sub-Saharan African countries, started nationwide access programs to ART, relying on the WHO guidelines for the public health approach. In the 15 years since implementation, the number of patients under ART in the country has therefore significantly increased, from less than 10,000 in early 2000s to almost 200,000 in 2017, representing a coverage of about 30% of the estimated number of HIV-infected individuals in the country [17]. Several studies have assessed the effectiveness of increasing access to ART, but they have generally focused on Yaoundé, the capital city, or other major cities, where ART programs were first implemented. All of these studies have been useful in providing information critical for evaluation of the national ART program in Cameroon, but no truly representative assessment at the national level has yet been reported.

In this study, we report the first nationally representative estimates of virological suppression and HIVDR in Cameroon for patients with median times on ART of either 17 (14–21) months or 53 (49–58) months. The 12–24 months assessment aims at evaluating virological failure during the first years of ART initiation, and the 48–60 months assessment evaluates the outcome later after ART initiation. Results indicate 72% viral suppression in the first group and 68% in the second group, representing 28% and 32% VF, respectively.

While no similar study with national coverage from sub-Saharan Africa has been published yet, results from studies conducted in various cities of the region and in Cameroon show variable rates of VF ranging from 10% to 30% [18], [19], [20]. Muwonga and colleagues reported 14.6% VF in patients on first-line ART in 4 cities in the Democratic Republic of Congo, after median times of 19 to 27 months on ART [13]; Messou and colleagues reported in Abidjan, Cote d'Ivoire, 20% and 25% VF among patients on first-line ART after 6 months and 12 months respectively [21]; a high rate of VF of 30.8% was reported for patients in Lomé, Togo, on first-line ART for one year (10–14 months) [10]; and we reported failure rates ranging from 2.9% to 20.6% after 12 months on first-line ART and 3.7% to 26% after 24 months on first-line ART, in a recent study involving 7 countries from west Africa and south-east Asia [9]. In Cameroon, results of one of the first clinical trials conducted in the country in 2004, reported viral suppression of 80% at week 24 after ART initiation, demonstrating efficacy of ART in a low-income country if adequate monitoring, including VL, is implemented [22]. Additional cross-sectional studies report VF rates ranging from 16.4% and 22.5% after 12 and 24 months ART respectively [11]; we reported in 2013, 18% VF after 36 months ART [23]; and recently, a study conducted in a semi-rural clinic close to Yaoundé reported 24% VF after 29 months on ART [24]. Combined, the significant heterogeneity of virological outcomes across sites and countries means that national estimates of VF cannot be simply extrapolated from studies not designed to be nationally representative.

Recent studies conducted in Cameroon, mostly in clinics located in the capital city Yaoundé, indicate improving virological outcomes at 12 months and 24 months, with around 10% to 15% VF [9], [20]. However, results of the present, nationally representative study are much more concerning (up to 30% failure) and indicate poor ART outcomes at the national level in Cameroon. Moreover, it should be noted that our results likely underestimate the prevalence of viral failure and HIVDR as outcomes are only measured among people who are retained in care and on ART and therefore likely to have better outcomes. Indeed, because of the cross-sectional design, we missed information on patients lost to follow-up and those who have died, which likely include additional failures [25]. Indeed, in the study conducted in a clinic in the capital city Yaoundé in 2013, Billong and colleagues reported lost to follow-up as a major programmatic issue and potentially associated with treatment failure and HIVDR [25]. A national assessment of HIVDR early warning indicators (HIVDR-EWI) conducted in 2015 also highlighted delays in drugs pick-up and drug stock-outs as additional major factors favoring emergence of HIVDR, and rural regions were more affected compared to urban regions [27]. Adherence to treatment is so far considered as a major factor associated with treatment failure and HIVDR and was reported in Cameroon as an important programmatic issue [20], [27], strengthening the necessity of routine access to viral load monitoring.

HIVDR was detected in 63% (ART 12–24) and in 88% (ART 48–60) of patients with VF, similar to results from other studies in sub-Saharan Africa [4], [18]. Importantly, we found the likelihood of developing HIVDR was much higher at rural sites compared to urban sites. Indeed, up to 23.2% of patients receiving ART in rural sites developed drug resistance compared to 14.0% in urban sites. Previous studies from rural regions, although limited to a few clinics, have reported similarly high frequencies of drug resistance, but also for VF [12], [24], [26]. Possible explanations include challenges to regularly access health care facilities, drug stock out, lack of adequate infrastructures and qualified personnel [27]. These results stress the urgent necessity of specific ART monitoring and management approaches for rural regions in sub-Saharan African.

Overall, in our study population considering both time points, 18% to 30% of patients on ART carried a drug resistant virus. This is truly alarming, especially for the 20% of patients on ART for only 12 to 24 months who already carry a resistant virus and will therefore require a switch to a less accessible second-line treatment that is harder to access and more costly. DRMs and mutation profiles correlated with ART regimens, with NRTI and NNRTI mutations predominating and almost no PI mutations. As reported in other studies, low genetic barrier mutations M184V and K103N were observed at high prevalence [5], [7]. However, contrary to previous studies conducted in Cameroon, we observed a high prevalence of K65R mutation, which is probably associated with the recent introduction of tenofovir in the first-line regimen. A recent study has reported worryingly high rates of tenofovir-associated drug resistance mutations in patients failing tenofovir-based first-line in sub-Saharan Africa [28]. We speculate that the risk of developing this mutation increases significantly in contexts with limited virological monitoring. Also, as drug resistance is not routinely assessed prior to ART initiation in Cameroon and in other low- and middle-income countries, as per WHO recommendations, we cannot exclude that some patients carried pre-treatment drug resistance mutations, including transmitted drug resistance, and are therefore at high risk of premature ART failure and emergence of drug resistance [29]. Considering our results as representing only newly acquired HIVDR may thus lead to over-estimation. As reported in previous studies, we identified several patients whose viruses accumulated many DRMs (up to nine mutations) [6], [7]. The fact that we found similar levels of accumulation in both study groups (ART 12–24 and ART 48–60), suggests that DMR accumulation in patients with virologic failure occurs early after treatment initiation, probably within the first two years of treatment initiation. This emphasizes the need for early adherence support and timely virological monitoring after treatment initiation.

This study has, however some limitations. The use of DBS to perform HIV VL has been reported as leading to some false-positive results due to pro-viral DNA contamination, especially for low-level VLs, < 5000 copies/ml [16]. This may have induced an over estimation of the VF. Moreover, the overall prevalence of drug resistance may have been underestimated, since we excluded individuals with VL ≥ 1000 copies/ml and with unsuccessful genotypes. Finally, exclusion of a few sites may have introduced bias in the national representativeness of results.

This study represents the first nationally representative estimate of viral suppression and HIVDR rate in Cameroon. The results indicate that achieving 90% viral suppression by 2020 as per UNAIDS objectives will require significant effort. Free access to VL testing is not yet available in Cameroon and in the light of these results, is now urgently needed. Implementation of standardized practices to ensure rapid switching to second-line ART can limit the accumulation of DRMs. At the global level, resistance to tenofovir is a concern and should be carefully monitored. Finally, first-line agents with higher genetic barriers to resistance as the new generation of integrase inhibitors represent good options in sub-Saharan Africa.

The following are the supplementary data related to this article.

Supplementary Table 1 Viral load and HIV drug resistance proportions in each clinic. Supplementary Table 1

Supplementary Table 2 HIV-1 drug resistance mutation profiles. Supplementary Table 2

## The Study Group

Sylvie Abia, Avelin Fobang Aghokeng, Silvia Bertagnolio, Dorothée Bessala, Christelle Butel, Corneluis Chebo, Oumarou Chifen, Amandine Cournil, John E. Ebonloe, Sabrina Eymard-Duvernay, Gaspary Fodjeu, Suzanne Izard, Brigitte Kamtie, Emmanuel Chia Kiawi, Charles Kouam Kouam, Charles Kouanfack, Nadine Lamare, Emilienne Mamang, Nadia Mandeng, Eyongetah Mbu, Bouba Mfokue, Jembia Joseph Mosoko, Bernard Nandjou, Mireille Mpoudi, Eitel Mpoudi-Ngole, Mariama Ndam, Anne Njom Nlend, Batam Nlend, Cecile Nouboué, Pierrette Omgba, Thierry Owono, Florant Oyono, Ida Penda, Elliot Raizes, Laetitia Serrano, Xavier Tchetnya, Christian Tchinou, Gaëlle Francine Tchouwa.

## Contributors

AFA, ER, SB, and GT worked on study design. GT, SE, AC, LS, and CB contributed on data collection, laboratory and data analysis. AFA, EM, ER contributed on data interpretation and writing. AFA was the study lead.

## Funding

This work was supported by the President's Emergency Plan for AIDS Relief (PEPFAR) through the US Centers for Disease Control and Prevention (CDC) under the terms of 1U01GH000755. The findings and conclusions in this paper are those of the authors and do not necessarily represent the official position of the funding agencies.

## Declaration of Interests

The authors declare no conflicts of interest.

## References

[1] UNAIDS (2014). Fast-track - ending the AIDS epidemic by 2030. http://www.unaids.org/sites/default/files/media_asset/JC2686_WAD2014report_en.pdf.

[2] WHO (2015). Guideline on when to start antiretroviral therapy and on pre-exposure prophylaxis for HIV. http://apps.who.int/iris/bitstream/10665/186275/1/9789241509565_eng.pdf.

[3] Bertagnolio S., Perno C.F., Vella S., Pillay D. (2013). The impact of HIV drug resistance on the selection of first- and second-line ART in resource-limited settings. J Infect Dis.

[4] Guichet E., Aghokeng A., Serrano L. (2016). Short communication: high viral load and multidrug resistance due to late switch to second-line regimens could be a major obstacle to reach the 90-90-90 UNAIDS objectives in sub-Saharan Africa. AIDS Res Hum Retroviruses.

[5] Wallis C.L., Mellors J.W., Venter W.D., Sanne I., Stevens W. (2010). Varied patterns of HIV-1 drug resistance on failing first-line antiretroviral therapy in South Africa. J Acquir Immune Defic Syndr.

[6] Barth R.E., Aitken S.C., Tempelman H. (2012). Accumulation of drug resistance and loss of therapeutic options precede commonly used criteria for treatment failure in HIV-1 subtype-C-infected patients. Antivir Ther.

[7] Boender T.S., Kityo C.M., Boerma R.S. (2016). Accumulation of HIV-1 drug resistance after continued virological failure on first-line ART in adults and children in sub-Saharan Africa. J Antimicrob Chemother.

[8] Sigaloff K.C., Ramatsebe T., Viana R., Wit T.F., Wallis C.L., Stevens W.S. (2011). Accumulation of HIV drug resistance mutations in patients failing first-line antiretroviral treatment in South Africa. AIDS Res Hum Retroviruses.

[9] Aghokeng A.F., Monleau M., Eymard-Duvernay S. (2014). Extraordinary heterogeneity of virological outcomes in patients receiving highly antiretroviral therapy and monitored with the World Health Organization public health approach in sub-Saharan Africa and Southeast Asia. Clin Infect Dis.

[10] Dagnra A.Y., Vidal N., Mensah A. (2011). High prevalence of HIV-1 drug resistance among patients on first-line antiretroviral treatment in Lome, Togo. J Int AIDS Soc.

[11] Kouanfack C., Montavon C., Laurent C. (2009). Low levels of antiretroviral-resistant HIV infection in a routine clinic in Cameroon that uses the World Health Organization (WHO) public health approach to monitor antiretroviral treatment and adequacy with the WHO recommendation for second-line treatment. Clin Infect Dis.

[12] Liegeois F., Vella C., Eymard-Duvernay S. (2012). Virological failure rates and HIV-1 drug resistance patterns in patients on first-line antiretroviral treatment in semirural and rural Gabon. J Int AIDS Soc.

[13] Muwonga J., Edidi S., Butel C. (2011). Resistance to antiretroviral drugs in treated and drug-naive patients in the Democratic Republic of Congo. J Acquir Immune Defic Syndr.

[14] Boerma R.S., Boender T.S., Bussink A.P. (2016). Suboptimal viral suppression rates among HIV-infected children in low- and middle-income countries: a meta-analysis. Clin Infect Dis.

[15] WHO (2014). Surveillance of HIV drug resistance in adults receiving ART (acquired HIV drug resistance). http://apps.who.int/iris/bitstream/10665/112802/1/9789241507196_eng.pdf.

[16] Monleau M., Aghokeng A.F., Eymard-Duvernay S. (2014). Field evaluation of dried blood spots for routine HIV-1 viral load and drug resistance monitoring in patients receiving antiretroviral therapy in Africa and Asia. J Clin Microbiol.

[17] UNAIDS (2017). Country factsheets CAMEROON-2016. http://www.unaids.org/en/regionscountries/countries/cameroon.

[18] Hosseinipour M.C., Gupta R.K., Van Zyl G., Eron J.J., Nachega J.B. (2013). Emergence of HIV drug resistance during first- and second-line antiretroviral therapy in resource-limited settings. J Infect Dis.

[19] Boender T.S., Sigaloff K.C., McMahon J.H. (2015). Long-term virological outcomes of first-line antiretroviral therapy for HIV-1 in low- and middle-income countries: a systematic review and meta-analysis. Clin Infect Dis.

[20] Meresse M., March L., Kouanfack C. (2014). Patterns of adherence to antiretroviral therapy and HIV drug resistance over time in the Stratall ANRS 12110/ESTHER trial in Cameroon. HIV Med.

[21] Messou E., Chaix M.L., Gabillard D. (2011). Association between medication possession ratio, virologic failure and drug resistance in HIV-1-infected adults on antiretroviral therapy in Cote d'Ivoire. J Acquir Immune Defic Syndr.

[22] Laurent C., Kouanfack C., Koulla-Shiro S. (2004). Effectiveness and safety of a generic fixed-dose combination of nevirapine, stavudine, and lamivudine in HIV-1-infected adults in Cameroon: open-label multicentre trial. Lancet.

[23] Aghokeng A.F., Kouanfack C., Eymard-Duvernay S. (2013). Virological outcome and patterns of HIV-1 drug resistance in patients with 36 months' antiretroviral therapy experience in Cameroon. J Int AIDS Soc.

[24] Boulle C., Guichet E., Kouanfack C. (2016). Virologic failure and human immunodeficiency virus drug resistance in rural Cameroon with regard to the UNAIDS 90-90-90 treatment targets. Open Forum Infect Dis.

[25] Billong S.C., Fokam J., Aghokeng A.F. (2013). Population-based monitoring of emerging HIV-1 drug resistance on antiretroviral therapy and associated factors in a sentinel site in Cameroon: low levels of resistance but poor programmatic performance. PLoS One.

[26] Taieb F., Aghokeng A.F., Eymard-Duvernay S. (2014). Challenges of antiretroviral treatment monitoring in rural and remote-access regions in Africa. AIDS Res Hum Retroviruses.

[27] Fokam J., Elat J.B., Billong S.C. (2015). Monitoring HIV drug resistance early warning indicators in Cameroon: a study following the revised World Health Organization recommendations. PLoS One.

[28] TenoRes Study Group (2016). Global epidemiology of drug resistance after failure of WHO recommended first-line regimens for adult HIV-1 infection: a multicentre retrospective cohort study. Lancet Infect Dis.

[29] Ceccarelli L., Salpini R., Moudourou S. (2012). Characterization of drug resistance mutations in naive and ART-treated patients infected with HIV-1 in Yaounde, Cameroon. J Med Virol.

