# Supplementary material for: Nationwide Estimates of Viral Load Suppression and Acquired HIV Drug Resistance in Cameroon
Source: eClinicalMedicine. 2018 Jul 4;1:21–7. doi: 10.1016/j.eclinm.2018.06.005 (PMC6537545; doi:10.1016/j.eclinm.2018.06.005)
Supplement: Supplementary Table 1 — Viral load and HIV drug resistance proportions in each clinic. [file mmc1.docx]

Supplementary Table 1. Viral load and HIV drug resistance proportions in each clinic.

| Site | Country region | N (1064) | n (796) | VL <1000 copies/ml |  | N (164) | n (113) | HIVDR proportion |
| --- | --- | --- | --- | --- | --- | --- | --- | --- |
| ART 12-24 | | | | | | | | |
|  |  |  |  |  |  |  |  |  |
| U_Regional_Limbe | South-west | 53 | 38 | 0,72 |  | 10 | 8 | 0,80 |
| R_District_Kumba | South-west | 62 | 38 | 0,61 |  | 14 | 12 | 0,86 |
| R_District_Muyuka | South-west | 29 | 19 | 0,66 |  | 8 | 7 | 0,88 |
| R_District_Abong_Mbang | East | 42 | 27 | 0,64 |  | 9 | 6 | 0,67 |
| R_UPEC_Bangante | West | 45 | 35 | 0,78 |  | 4 | 3 | 0,75 |
| R_District_Mbouda | West | 44 | 34 | 0,77 |  | 4 | 3 | 0,75 |
| U_Police_Bafoussam | West | 25 | 18 | 0,72 |  | 6 | 4 | 0,67 |
| U_Regional_Bafoussam | West | 72 | 55 | 0,76 |  | 8 | 7 | 0,88 |
| U_District_Nylon | Littoral | 49 | 45 | 0,92 |  | 3 | 1 | 0,33 |
| U_Laquintinie | Littoral | 45 | 32 | 0,71 |  | 10 | 7 | 0,70 |
| U_CMA_Soboum | Littoral | 60 | 53 | 0,88 |  | 2 | 1 | 0,50 |
| U_District_Deido | Littoral | 71 | 46 | 0,65 |  | 16 | 7 | 0,44 |
| U_General_Douala | Littoral | 6 | 3 | 0,50 |  | 3 | 2 | 0,67 |
| R_Bafia | Center | 51 | 30 | 0,59 |  | 9 | 3 | 0,33 |
| U_CNPS | Centre | 39 | 35 | 0,90 |  | 3 | 2 | 0,67 |
| U_Central_Yaounde* | Centre | 56 | 51 | 0,91 |  | 5 | 5 | 1,00 |
| U_Militaire_Yaounde | Centre | 39 | 35 | 0,90 |  | 4 | 4 | 1,00 |
| U_District_Cite_Verte | Centre | 68 | 60 | 0,88 |  | 7 | 7 | 1,00 |
| U_Universite_Yaounde | Centre | 61 | 55 | 0,90 |  | 6 | 5 | 0,83 |
| U_District_Mbalmayo | South | 62 | 34 | 0,55 |  | 15 | 6 | 0,40 |
| U_District_Ambam | South | 18 | 15 | 0,83 |  | 2 | 1 | 0,50 |
| U_Regional_Bamenda | North-west | 26 | 15 | 0,58 |  | 5 | 3 | 0,60 |
| R_Nkambe | North-west | 41 | 23 | 0,56 |  | 11 | 9 | 0,82 |
|  |  |  |  |  |  |  |  |  |
|  |  |  |  |  |  |  |  |  |
|  |  |  |  |  |  |  |  |  |
| ART 48-60 |  | N (388) | n (267) |  |  | N (67) | n (59) |  |
| U_Bertoua | East | 57 | 36 | 0,63 |  | 8 | 8 | 1,00 |
| U_Ebolowa | South | 42 | 24 | 0,57 |  | 10 | 9 | 0,90 |
| U_Central_Yaounde | Centre | 54 | 47 | 0,87 |  | 6 | 6 | 1,00 |
| U_Laquintinie | Littoral | 60 | 45 | 0,75 |  | 7 | 4 | 0,57 |
| U_Regional_Bafoussam | West | 83 | 66 | 0,80 |  | 4 | 3 | 0,75 |
| U_Regional_Bamenda | North-west | 27 | 10 | 0,37 |  | 15 | 14 | 0,93 |
| U_Regional_Limbe | South-west | 65 | 39 | 0,60 |  | 17 | 15 | 0,88 |

U: Urban; R: rural

* This site was sampled two times according to random selection results.
