# Supplementary material for: Nationwide Estimates of Viral Load Suppression and Acquired HIV Drug Resistance in Cameroon
Source: eClinicalMedicine. 2018 Jul 4;1:21–7. doi: 10.1016/j.eclinm.2018.06.005 (PMC6537545; doi:10.1016/j.eclinm.2018.06.005)
Supplement: Supplementary Table 2 — HIV-1 drug resistance mutation profiles. [file mmc2.docx]

Supplementary Table 2. HIV-1 drug resistance mutation profiles

| Mutation profiles | ART-1 *(12-24M)*  *(n=109)* | ART-2 *(48-60M)*  *(n=58)* |
| --- | --- | --- |
| K103N,M184V | 11 | 6 |
| K103N | 6 | 5 |
| K103N,M184I | 3 | 0 |
| K65R,L100I,K103N,M184V | 3 | 0 |
| M184V,G190A | 3 | 1 |
| V106A,M184V | 3 | 0 |
| D67N,K70E,M184V,Y188L,T215F | 2 | 0 |
| K103N,G190A | 2 | 0 |
| K103N,M184V,P225H | 2 | 1 |
| K103N,Y181C,M184V | 2 | 0 |
| K65R,K103N,Y115F,Y181C,M184V | 2 | 0 |
| K65R,Y181C,M184I | 2 | 0 |
| Y181C,M184V | 2 | 1 |
| D67N,K103N,M184V | 1 | 0 |
| D67N,K70R,K101E,M184V,G190A,T215I,K219E | 1 | 0 |
| D67N,K70R,K101P,K103S,M184V,T215FIS,K219E | 1 | 0 |
| D67N,K70R,K103N,M184V,G190A,T215F,K219E | 1 | 0 |
| D67N,K70R,K103N,M184V,T215Y,K219E,P225H | 1 | 0 |
| D67N,K70R,K103N,Y181C,M184V,G190A,T215F,K219E | 1 | 0 |
| D67N,K70R,L74I,K103N,M184V,G190E,T215F,K219Q | 1 | 0 |
| D67N,K70R,M184V,G190A,T215Y,K219E | 1 | 0 |
| D67N,K70R,M184V,Y188L,G190A,T215Y,K219E | 1 | 0 |
| D67N,K70R,V106A,M184V,T215F,K219Q | 1 | 0 |
| D67N,K70R,Y181C,M184V,G190A,T215I,K219E | 1 | 0 |
| D67N,K70R,Y181C,M184V,T215F,K219Q | 1 | 0 |
| D67N,K70R,Y181C,M184V,T215I,K219E | 1 | 0 |
| G190A | 1 | 0 |
| K101E | 1 | 0 |
| K101E,K103N | 1 | 0 |
| K101E,M184V,G190A,K219E | 1 | 0 |
| K101E,M184V,G190A,P225H | 1 | 0 |
| K103N,M184I,P225H,M230L | 1 | 0 |
| K103N,M184V,G190A | 1 | 0 |
| K103N,M184V,T215F | 1 | 0 |
| K103N,M184V,T215SY | 1 | 0 |
| K103N,Y181C,M184V,G190A,T215F | 1 | 0 |
| K65R,K101E,Y181C,M184V,G190A | 1 | 0 |
| K65R,K101E,Y181C,M184V,G190A,K219E | 1 | 0 |
| K65R,K103N,M184I,M230L | 1 | 0 |
| K65R,K103N,M184V | 1 | 2 |
| K65R,K103N,Y115F | 1 | 0 |
| K65R,K103N,Y115F,M184V,Y188L | 1 | 0 |
| K65R,K103N,Y115F,Y188C | 1 | 0 |
| K65R,K103N,Y181C,G190A | 1 | 0 |
| K65R,K103N,Y181C,M184V | 1 | 0 |
| K65R,K103N,Y181C,M184V,K219E | 1 | 0 |
| K65R,K70E,K103N,Y181C,M184V | 1 | 0 |
| K65R,L100I,K103N | 1 | 0 |
| K65R,L100I,K103N,M184I,M230L | 1 | 0 |
| K65R,L74I,L100I,K103N,M184V,K219E | 1 | 0 |
| K65R,M184V,Y188L | 1 | 0 |
| K65R,V106A,Y181C,M184I,P225H | 1 | 0 |
| K65R,V106M,M184V,Y188C | 1 | 0 |
| K65R,Y181C,M184I,G190A,M230L | 1 | 0 |
| K65R,Y181C,M184I,G190S | 1 | 1 |
| K65R,Y181C,M184V | 1 | 0 |
| K65R,Y181C,M184V,G190A | 1 | 0 |
| K65R,Y181C,M184V,G190A,K219E | 1 | 0 |
| K65R,Y181I,G190E | 1 | 0 |
| K65R,Y181V,M184V | 1 | 0 |
| K70E,K103N,M184V,P225H | 1 | 0 |
| K70E,K103N,M184V,Y188L | 1 | 0 |
| K70E,L74I,K103N,M184V,P225H | 1 | 0 |
| K70E,M184V,G190A,P225H | 1 | 1 |
| K70E,Y181C,M184IV,G190A | 1 | 0 |
| K70E,Y181C,M184V | 1 | 0 |
| L74I,K103N,M184I | 1 | 0 |
| L74I,K103N,M184V,K219R,P225H | 1 | 0 |
| L74V,K101E,Y181C,M184V,G190S | 1 | 0 |
| M41L,D67N,L74I,K103N,M184V,L210W,T215Y,K219Q | 1 | 0 |
| M41L,D67N,Y181C,M184V,T215Y | 1 | 0 |
| M41L,K103S,M184V,G190A,L210W,T215Y | 1 | 0 |
| M41L,K65R,M184V,Y188L | 1 | 0 |
| M41L,V75M,M184V,Y188L,L210W,T215FY | 1 | 0 |
| T215I | 1 | 0 |
| V106A | 1 | 0 |
| V75M,K101E,Y181C,M184V,G190A,T215Y,K219E | 1 | 0 |
| Y181C,M184IV,Y188L | 1 | 0 |
| Y181C,M184V,P225H | 1 | 0 |
| D67N,K70R,K103N,V106A,M184V,T215F,K219Q | 0 | 1 |
| D67N,K70R,M184V,Y188L,T215F,K219Q | 0 | 1 |
| D67N,K70R,M184V,Y188L,T215I,K219E | 0 | 1 |
| D67N,K70R,Y181C,M184V,K219E | 0 | 1 |
| D67N,K70R,Y181C,M184V,K219Q | 0 | 1 |
| D67N,K70R,Y181C,M184V,T215Y | 0 | 1 |
| D67N,T69D,K70R,K103N,Y181C,M184V,G190A,T215I,K219E | 0 | 1 |
| D67N,V106A | 0 | 1 |
| K101E,M184V,G190S | 0 | 1 |
| K103N,M184V,Y188L,T215I | 0 | 1 |
| K103N,V106M,M184IV | 0 | 1 |
| K103N,Y181C,M184V,G190A | 0 | 1 |
| K65R,D67G,K103N,Y115F,Y188C | 0 | 1 |
| K65R,K103N,M184V,P225H | 0 | 1 |
| K65R,K103N,Y115F,Y181C,M184V,G190A | 0 | 1 |
| K65R,K103N,Y181C | 0 | 1 |
| K65R,K103N,Y181C,M184I | 0 | 2 |
| K65R,K103N,Y181C,M184V,G190A | 0 | 1 |
| K65R,L100I,K103N,M184V,P225H | 0 | 1 |
| K65R,M184V,Y188L,G190A | 0 | 1 |
| K70E,K103N,M184V,M230L | 0 | 1 |
| K70R,V106A,M184V,K219R | 0 | 1 |
| L74I,K103N,M184V,L210W,T215F | 0 | 1 |
| L74I,K103N,Y181C,M184V,T215F | 0 | 1 |
| L74I,K103NS,M184V,G190A,P225H | 0 | 1 |
| M184V,G190S | 0 | 1 |
| M184V,Y188L | 0 | 1 |
| M184V,Y188L,T215Y | 0 | 1 |
| M41L,D67G,K70R,V75M,F77L,Y181C,M184V,T215F,K219E | 0 | 1 |
| M41L,D67N,V75M,K103N,M184V,L210W,T215FY,K219E,P225H | 0 | 1 |
| M41L,K103N,M184V,L210W,T215Y | 0 | 1 |
| M41L,K103N,M184V,T215Y | 0 | 1 |
| M41L,K65R,K101E,Y115F,Y181C,M184V,G190A | 0 | 1 |
| M41L,L74I,K103N,M184V,L210W,T215Y | 0 | 1 |
| M41L,L74V,K101P,K103N,M184V,T215Y | 0 | 1 |
| M41L,L74V,Y181C,M184V,L210W,T215F,M230L | 0 | 1 |
| M41L,M184V,Y188L,T215FY | 0 | 1 |
| M41L,T69D,K70R,V75M,K103N,M184V,T215F,K219Q,M230L | 0 | 1 |
| Y181C,M184V,T215F | 0 | 1 |
